# Human stem cell-derived hepatocyte-like cells support Zika virus replication and provide a relevant model to assess the efficacy of potential antivirals

**DOI:** 10.1371/journal.pone.0209097

**Published:** 2018-12-19

**Authors:** Tine Tricot, Nicky Helsen, Suzanne J. F. Kaptein, Johan Neyts, Catherine M. Verfaillie

**Affiliations:** 1 Stem Cell Institute, University of Leuven (KU Leuven), Leuven, Belgium; 2 KU Leuven, Department of Microbiology and Immunology, Rega Institute for Medical Research, Laboratory of Virology and Chemotherapy, Leuven, Belgium; University of Tennessee Health Science Center, UNITED STATES

## Abstract

Zika virus (ZIKV) infection during pregnancy has been extensively linked to microcephaly in newborns. High levels of ZIKV RNA were, however, also detected in mice and non-human primates in organs other than the brain, such as the liver. As ZIKV is a flavivirus closely related to the dengue and yellow fever virus, which are known to cause hepatitis, we here examined whether human hepatocytes are susceptible to ZIKV infection. We demonstrated that both human pluripotent stem cell (hPSC)-derived hepatocyte-like cells (HLCs) and the Huh7 hepatoma cell line support the complete ZIKV replication cycle. Of three antiviral molecules that inhibit ZIKV infection in Vero cells, only 7-deaza-2’-*C*-methyladenosine (7DMA) inhibited ZIKV replication in hPSC-HLCs, while all drugs inhibited ZIKV infection in Huh7 cells. ZIKV-infected hPSC-HLCs but not Huh7 cells mounted an innate immune and NFκβ response, which may explain the more extensive cytopathic effect observed in Huh7 cells. In conclusion, ZIKV productively infects human hepatocytes *in vitro*. However, significant differences in the innate immune response against ZIKV and antiviral drug sensitivity were observed when comparing hPSC-HLCs and hepatoma cells, highlighting the need to assess ZIKV infection as well as antiviral activity not only in hepatoma cells, but also in more physiologically relevant systems.

## Introduction

Zika virus (ZIKV) is a mosquito-borne flavivirus mostly transmitted by *Aedes* mosquitos. Cases of sexual transmission and transmission via blood transfusion have, however, also been described [1–4]. Most ZIKV-infected patients are asymptomatic or present with mild clinical symptoms such as rash, conjunctivitis and arthralgia [5,6]. A major public health concern is, however, the link between ZIKV infection and abnormalities during fetal development, and more specifically brain development. The virus has been detected in the amniotic fluid of pregnant women and in the brain tissue of fetuses with microcephaly [7,8]. Furthermore, the African ZIKV MR766 strain was reported to infect human induced pluripotent stem cell (hiPSC)-derived cortical neuroprogenitors (NPCs), causing increased NPC apoptosis, as well as hiPSC-derived cortical and motor neurons [9–12]. Additionally, several newborns with microcephaly were also diagnosed with Guillain-Barré syndrome and eye defects [13,14]. It was hypothesized that the eye defects are due to endogenous hypervitaminosis as a result of ZIKV-induced hepatitis and liver damage [15].

Recently, others and we reported on mouse models to study ZIKV infection. In these mice, high viral loads were not only detected in the brain and spinal cord but also in the kidney, spleen, liver and testes [16–18]. The dynamics of ZIKV infection, replication and shedding have also been studied in pregnant ZIKV-infected rhesus and cynomolgus macaques [19–21]. ZIKV RNA was detected in the fetal brain and liver, in the placenta and in the maternal brain, eyes, liver and spleen upon delivery [22]. Furthermore, elevated alanine aminotransferase (ALT) and aspartate aminotransferase (AST) levels were detected in the blood of ZIKV-infected macaques, indicating liver damage [21]. Of note, a case report published in 1954 described the occurrence of liver damage and jaundice in two patients with elevated ZIKV antibodies [23]. Interestingly, the association between ZIKV infection and liver injury was also demonstrated in a more recent case report in 2017. Both, ALT and AST levels were significantly increased and blood coagulation parameters were remarkably altered [24]. However, to the best of our knowledge, there are no reports of any experimental demonstration of ZIKV replication in human hepatocytes.

Both dengue virus (DENV) and yellow fever virus (YFV), which together with ZIKV belong to the family of the *Flaviviridae*, are known to infect human hepatocytes [25–27]. Unfortunately, research into the permissiveness of human hepatocytes for ZIKV is complicated by the fact that the availability of primary human hepatocytes (PHHs) is limited and that they rapidly dedifferentiate in culture, losing their hepatocyte characteristics [28,29]. Others and we previously demonstrated that human pluripotent stem cell (hPSC)-derived hepatocyte-like cells (HLCs) support the complete replication cycle of hepatotropic viruses, including hepatitis E virus (HEV) [30], hepatitis B virus (HBV) [31–33], hepatitis C virus (HCV) [34–37] and DENV [38]. Given the fact that ZIKV can infect hepatocytes in mice and non-human primates, and the fact that other *Flaviviridae* are known to infect human hepatocytes, we here explored whether hPSC-HLCs are susceptible to ZIKV infection and, hence, could be used as a model to test antiviral approaches for ZIKV. We demonstrate that hPSC-HLCs as well as hepatoma cell line Huh7 support the complete ZIKV life cycle, including entry, replication and production of infectious virions. Interestingly, we also observed a significant difference, both in the innate immune response mounted against ZIKV in the two models, and in drug sensitivity of ZIKV replication.

## Materials and methods

### Cell cultures

The human embryonic stem cell (hESC) line H9 and the hiPSC line were purchased from WiCell Research Institute (Madison, US) and Sigma-Aldrich (Saint Louis, MO), respectively. Both hPSC lines were maintained on human matrigel (BD Biosciences, San Jose, CA) coated plates in E8 Flex medium (Gibco) in a humidified 5% CO_2_ incubator at 37°C. hPSCs were differentiated towards HLCs following a previously published 20-day differentiation protocol with minor adjustments [30,39]: namely, dimethyl sulfoxide (DMSO) at a concentration of 0.6% was added from day 0 until day 12 and increased to 2% from day 12 of differentiation onwards until the end of the hepatocyte differentiation. The human hepatoma cell lines Huh7 (kindly provided by Ralf Bartenschlager, University of Heidelberg, Germany) and Huh7.5 (ATCC, Virginia, US) were maintained in Dulbecco’s modified Eagle’s medium (DMEM) (Gibco) supplemented with 10% fetal bovine serum (FBS) (Gibco), 1% non-essential amino acids (NEAA) (Gibco) and 1% penicillin-streptomycin in a humidified 5% CO_2_ incubator at 37°C.

### Virus

Infections were performed using the ZIKV strain MR766 (Rhesus/1947/Uganda) (obtained from the European Virus Archive (EVA)) and the PRVABC59 clinical isolate (obtained from the World Reference Center for Emerging Viruses and Arboviruses (WRCEVA), through the University of Texas Medical Branch (UTMB) at Galveston, USA). Viral stocks were generated as previously described [16].

### Virus inoculation

On day 16 of hepatocyte differentiation, hPSC-HLCs were inoculated with either 0.02% or 2% ZIKV MR766 stock (viral titer: 1x10^3^ PFU/mL) or 0.0125% ZIKV PRVABC59 stock (viral titer: 1.6x10^5^ PFU/mL) in hepatocyte differentiation medium and incubated for 6h at 37°C in a 5% CO_2_ humidified incubator. Following incubation, cells were washed extensively with PBS and fresh hepatocyte differentiation medium was added. Half of the hepatocyte medium was replaced every other day and supernatant was collected 2, 4 and 6 days post infection (d pi). Cells were lysed with 350μL RLT buffer at 4 and 6d pi (Qiagen, Hilden). Infection of Huh7 cells and Huh7.5, a derivative clone, were performed similarly. Mock infection was performed as a negative control.

### Inhibition experiments

7-deaza-2'-*C*-methyl-D-adenosine (7DMA) and 2’-*C*-methylcytidine (2’CMC) were purchased from Carbosynth (Berkshire, UK). T705 (6-fluoro-3-hydroxy-2-pyrazinecarboxamide; Favipiravir) was obtained from BOC Sciences (New York, USA). ZIKV-infected hPSC-HLCs and Huh7 cells were treated with 3 different concentrations (7DMA: 10, 30 and 90μM; 2’CMC: 5, 15 and 45μM; T705: 25, 75 and 225μM) of each compound, based on the EC_50_ determined in Vero cells (African Green monkey kidney cells; ECACC) as previously published [16]. 7DMA, 2’CMC and T705 were added to the medium from viral inoculation until the end of the infection experiments.

### RNA isolation and RT-qPCR

Viral RNA was extracted from 150μL supernatant using the Nucleospin RNA virus kit (Macherey-Nagel, Düren) following the manufacturer’s protocol. Intracellular RNA was isolated by means of the RNeasy Mini kit (Qiagen). RT-qPCR was performed to analyze viral RNA levels as previously described [16]. Viral RNA levels in the lysates were normalized for total RNA content, which was measured by spectrometry (Nanodrop ND-1000, Thermo Fischer Scientific). For gene expression analysis, cDNA synthesis was performed using the Superscript III First-Strand synthesis kit (Invitrogen). Following cDNA synthesis, qPCR was performed with the Platinum SYBR green qPCR supermix-UDG kit (Invitrogen) using the ViiA7 Real-Time PCR instrument (Thermo Fischer Scientific, Waltham, US).

### Immunofluorescence staining

Cells were fixed with 4% paraformaldehyde 4d pi and washed with PBS, permeabilized with 0.2% Triton-X in PBS (0.2% PBST) and blocked with 5% donkey serum in 0.2% PBST. The cells were stained overnight at 4°C with anti-HNF4α (Ab41898, Abcam), anti-Flavivirus Group Antigen (clone D1-4G2-4-1, Millipore), anti-ZIKV NS3 (kindly provided by Andres Merits, Institute of Technology, University of Tartu, Estonia), anti-albumin (ALB; A0001, Dako), anti-α-fetoprotein (AFP; A008, Dako), anti-sodium taurocholate cotransporting peptide (NTCP; HPA042727, Sigma-Aldrich) and anti-Caspase 3 (active, cleaved form) (AB3623, Millipore) antibodies. Afterwards, the cells were incubated for 30 min at room temperature with the appropriate secondary antibodies and Hoechst (Sigma-Aldrich). Images were taken using the AxioimagerZ.1 fluorescence microscope (Carl Zeiss Inc.). Appropriate isotype control antibodies were used in all immunofluorescence staining experiments.

### Albumin ELISA

Enzyme-linked immunosorbent assay (ELISA) for albumin was performed following the manufacturer’s protocol (Bethyl, Montgomery, TX). At day 20 of HLC differentiation, supernatant was collected and incubated with a primary albumin antibody for 60 min at room temperature. Afterwards an HRP-detection antibody was added for 60 min at room temperature followed by an incubation with TMB-peroxidase solution for 15 min in the dark. The reaction was stopped by the addition of a 0.18M H2SO4 solution. The absorbance was measured at a wave length of 450nm.

### Re-infection assay

hESC-HLCs were inoculated with supernatant derived from ZIKV-infected hESC-HLCs (1:10 dilution), which was harvested at 6d pi. After incubation for 6h at 37°C in a 5% CO_2_ humidified incubator, the inoculum was removed and cells were washed extensively with PBS. Supernatant was collected 2d pi and 4d pi. On 4d pi, cells were lysed with 350μl RLT buffer (Qiagen). Viral RNA was quantified by RT-qPCR.

#### Plaque assay

4x10^5^ Baby Hamster Kidney (BHK) cells were seeded in a 12-well plate in 10% growth medium (DMEM with 10% FBS). Viral dilutions of supernatant harvested at 6d pi (1:10–1:50–1:250–1:1250) were prepared using 2% BHK medium (MEM REGA3 + 2% fetal calf serum). The cells were incubated for 2 hours with the virus dilutions in a humidified 5% CO_2_ incubator at 37°C. The virus dilutions were removed and a 0.8% carboxymethyl cellulose (CMC; Sigma Aldrich) overlay was added to the wells. After 1 week, the BHK cells were fixed and the samples were incubated for 5 min in 1% Crystal Violet (Sigma Aldrich), after which the cells were thoroughly washed with water and dried.

### MTS assay

hPSC-HLCs and Huh7 cells were seeded and differentiated in a 96-well format. On day 16 of hepatocyte differentiation, cells were infected with ZIKV. Six days post infection, medium was removed from the ZIKV-infected hPSC-HLCs and Huh7 cells. 100μL 3-(4,5-dimethylthiazol-2-yl)-5-(3-carboxymethoxyphenyl)-2-(4-sulfophenyl)-2*H*-tetrazolium/phenazinemethosulfate (MTS/PMS; Promega, Leiden, The Netherlands) was added to the cells. After 1h30min incubation at 37°C in a 5% CO_2_ humidified incubator, the optical density (OD) was determined at 498nm using the Tecan Safire^2 TM^. The % cell survival was calculated using the following formula: % survival = 100 x (OD_infected_/OD_uninfected_).

### Statistical analysis

Data are shown as mean ± SEM and analyzed by the two-tailed Student’s t test. p-values < 0.05 (*), p < 0.01 (**) and p < 0.001 (***) were considered statistically significant.

## Results

### Stem cell-derived hepatocyte-like cells and Huh7 cells are susceptible to ZIKV infection

**Fig 1:**
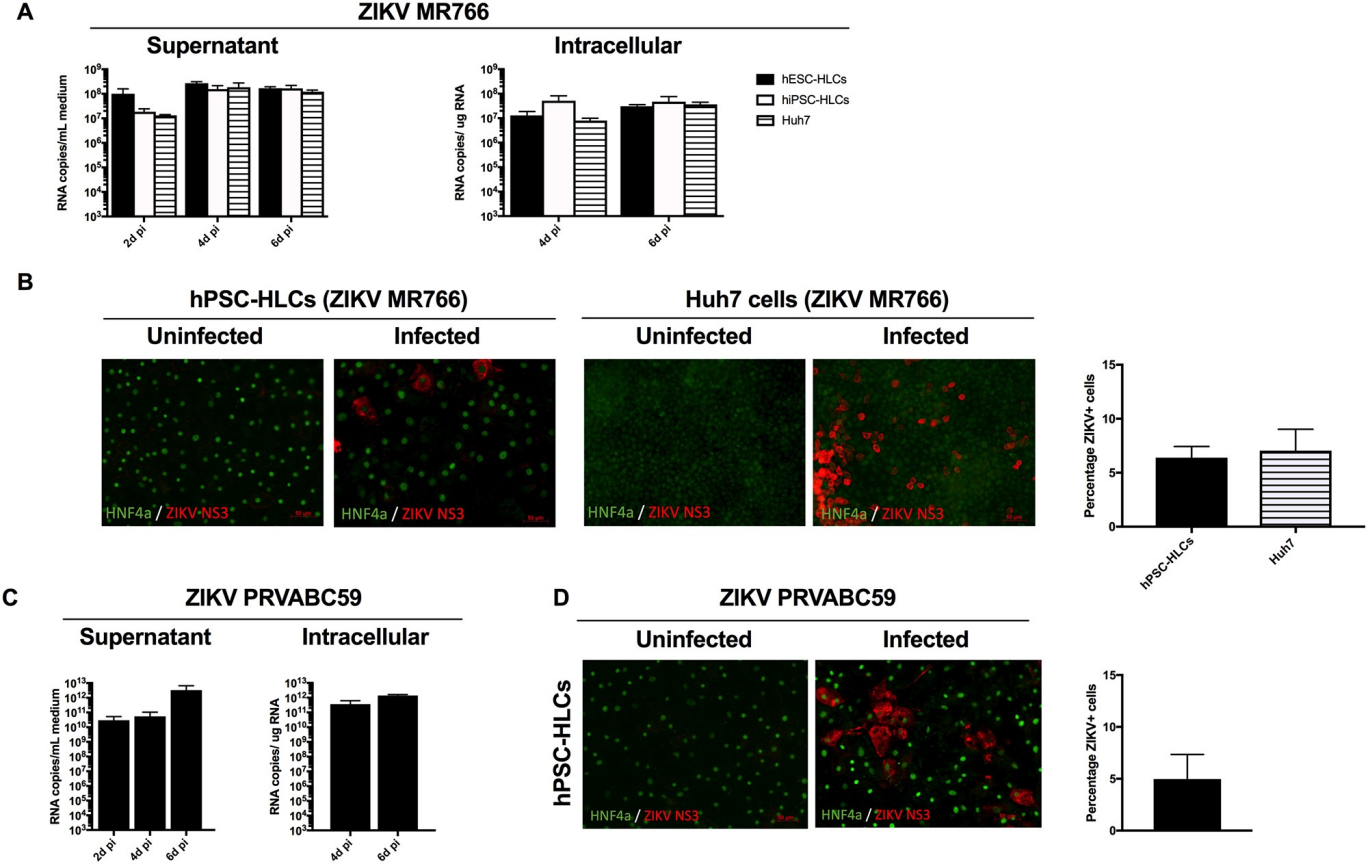
hPSC-HLCs and Huh7 were susceptible to ZIKV infection. (A) Infection of hPSC-HLCs and Huh7 cells using a high ZIKV MR766 inoculum. RT-qPCR analysis was used to quantify the viral RNA levels in the supernatant and cellular lysates (intracellular) (d pi = days post infection) (n = 3). (B) *Left*: Immunofluorescence staining performed at 4d pi for HNF4α and ZIKV NS3 in hPSC-HLCs and Huh7 cells infected with the MR766 strain. Images are representative of three independent experiments (Scale bar = 50μM). *Right*: Quantification of the immunofluorescence staining. (C) Infection of hPSC-HLCs with the Asian PRVABC59 strain. RT-qPCR analysis was used to quantify the viral RNA levels in the culture supernatant and cellular lysates (intracellular) (d pi = days post infection) (n = 3). (D) *Left*: Immunofluorescence staining at d4 pi for HNF4α and ZIKV NS3 in hPSC-HLCs and Huh7 cells infected with the PRVABC59 strain. Images are representative of three independent experiments (Scale bar = 50μM). *Right*: Quantification of immunofluorescence staining. All data are shown as mean±SEM.

To study the susceptibility of human hepatocytes to ZIKV infection, hESCs and hiPSCs were differentiated towards HLCs, that homogenously express different hepatocyte markers such as AFP, ALB, HNF4α and NTCP and secrete ALB (S1A–S1C Fig) [39]. On day 16 of hepatocyte differentiation, hPSC-progeny were infected with the African ZIKV MR766 strain (with an MOI of 8x10^-5^ (further described as high MR766 inoculum) or an MOI of 8x10^-7^ (further described as low MR766 inoculum)). We also infected the Huh7 hepatoma cells as a control. ZIKV RNA levels were quantified by RT-qPCR in culture supernatant on day 2, 4 and 6d pi and in cellular lysates on 4 and 6d pi. Infection efficiency upon infection with the high MR766 inoculum was comparable between ZIKV-infected hPSCs-HLCs and Huh7 cells, with 10^8^−10^9^ viral RNA copies/mL in the supernatant and 10^8^−10^9^ viral RNA copies/μg in cellular lysates at 2d pi (Fig 1A). Infection levels seen in hPSC-HLCs appeared to be higher compared to previously published ZIKV infection levels found in hPSC-motor and cortical neuron progeny [12], suggesting a higher replication potential of ZIKV in the stem cell-derived hepatocytes compared to the stem cell-derived neurons. ZIKV infection of hPSC-HLCs and Huh7 cells was confirmed on 4d pi by immunofluorescence staining for ZIKV NS3 and the liver-enriched transcription factor HNF4α, demonstrating 6.4 ± 1.0% and 7.1 ± 2.0% ZIKV-infected hPSC-HLCs and Huh7 cells, respectively (Fig 1B). Additionally, we infected hPSC-HLCs and Huh7 cells with a 100-fold lower MR766 ZIKV inoculum (i.e. low MR766 inoculum), since extremely high levels of RNA copies in the supernatant and lysates were observed as early as 2d pi. RNA copies in the supernatant and lysates of hPSC-HLCs and Huh7 were measured at 2, 4 and 6d pi. Replication efficiency at 2d pi in hPSCs-HLCs was approximately 10^6^ viral RNA copies/mL or viral RNA copies/μg in the supernatant or cellular lysates, respectively. The replication efficiency increased to approximately 10^8^ viral RNA copies/mL or /μg at 4d pi, which was similar to the replication efficiency when using the high MR766 inoculum (S1D Fig). By contrast, the low MR766 inoculum was not sufficient to infected Huh7 cells, suggesting that hPSC-HLCs are more susceptible to ZIKV infection than Huh7 cells.

Next, we tested whether the recent clinical strain PRVABC59 from the Asian lineage [7,40,41], which is linked to an increased risk of microcephaly in newborns in Brazil, also caused a productive infection in hPSC-HLCs. Replication of the Asian ZIKV strain (MOI of 8x10^-5^) in hPSC-HLCs was confirmed by measuring ZIKV RNA levels in culture supernatant and cellular lysates, and by immunofluorescence staining for ZIKV NS3 (Fig 1C and 1D). Strain PRVABC59 replicated at an efficiency of 10E^11^-10E^12^ viral RNA copies/mL or viral RNA copies/μg in the supernatant or cellular lysates, respectively, indicating that the Asian ZIKV strain appeared to be more infectious than the African ZIKV strain (Fig 1A). However, immunofluorescence staining for ZIKV NS3 antigen showed that the infection rate between MR766 ZIKV-infected hPSCs-HLCs and PRVABC59 ZIKV-infected hPSC-HLCs was comparable: 6.4 ± 1.0% and 5.0 ± 2.4% of the HLCs were ZIKV NS3 positive, respectively (Fig 1B and 1D).

### Differences in the activity of ZIKV antivirals in HLCs and Huh7 cells

**Fig 2:**
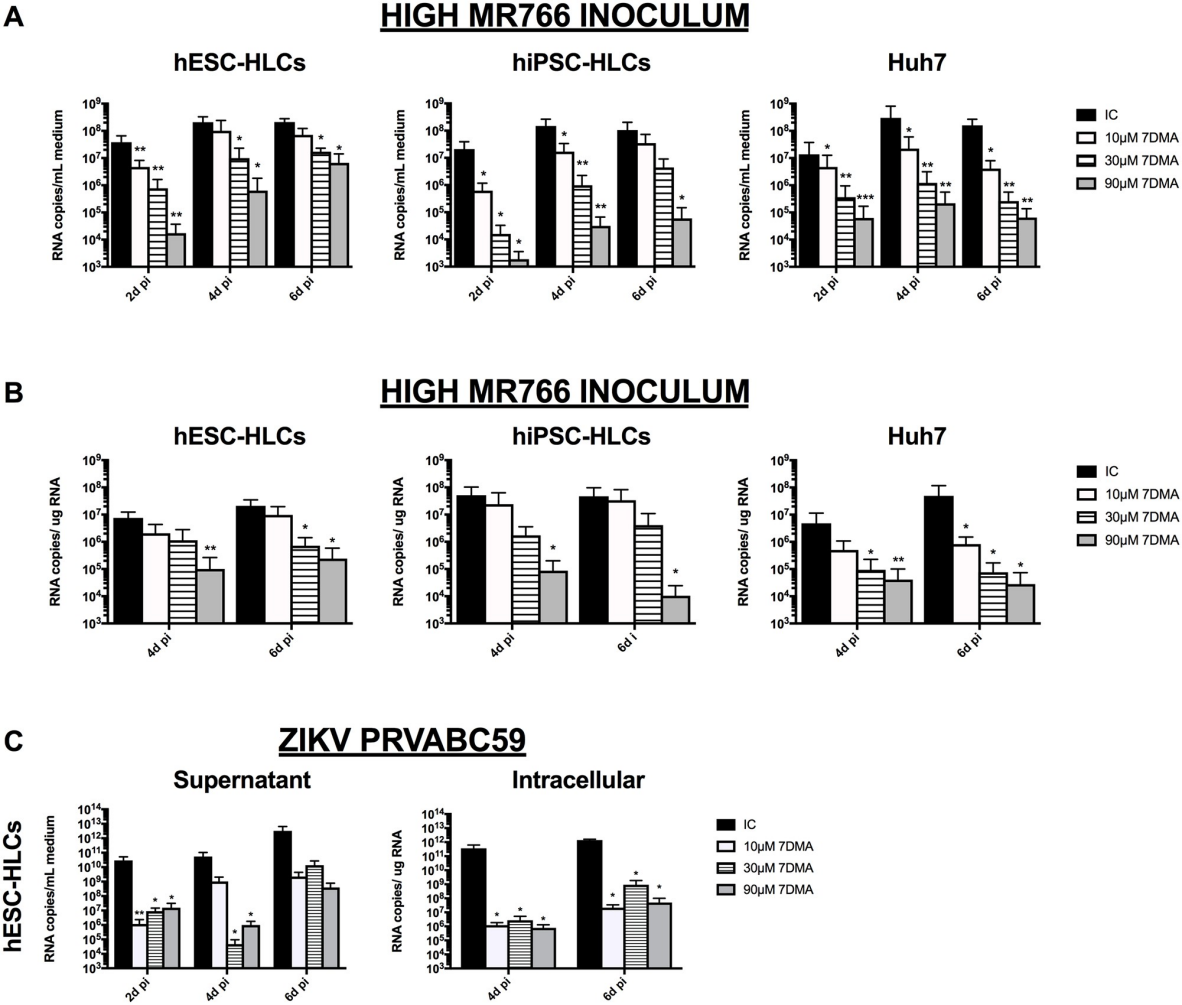
7DMA delayed ZIKV infection in hPSC-HLCs and Huh7 hepatoma cells. (A) RT-qPCR analysis of supernatant from hESC-HLCs, hiPSC-HLCs and Huh7 cells infected with a high inoculum of ZIKV MR766, in the presence of increasing concentrations of 7DMA (10μM—90μM) (IC = infected cells) (n = 3; *: p<0.05). (B) RT-qPCR analysis of cellular lysates of hESC-HLCs, hiPSC-HLCs and Huh7 cells, infected with a high inoculum of ZIKV MR766, in the presence of increasing concentrations of 7DMA (10μM—90μM) (IC = infected cells) (n = 3; *: p<0.05). (C) RT-qPCR analysis of supernatant and cellular lysates of hESC-HLCs, hiPSC-HLCs and Huh7 cells infected with the PRVABC59 strain, in the presence of increasing concentrations of 7DMA (10μM—90μM) (IC = infected cells) (n = 3; *: p<0.05). All data are shown as mean±SEM.

We next tested if ZIKV infection in hPSC-HLCs could be inhibited by specific antiviral compounds. We previously demonstrated that ZIKV replication in Vero cells is inhibited by three viral RNA-dependent RNA polymerase inhibitors, i.e. 7-deaza-2'-*C*-methyladenosine (7DMA), 2’-*C*-methylcytidine (2’CMC), and 6-fluoro-3-hydroxy-2-pyrazinecarboxamide (Favipiravir; T705). Moreover, 7DMA was also shown to delay ZIKV-induced mortality in mice and inhibited ZIKV-induced cytopathic effect in hiPSC-derived cortical and motor neurons [12,16]. 7DMA and 2’CMC were initially developed as polymerase inhibitors against the hepatitis C virus [42,43]. T-705 is a broad-spectrum inhibitor with antiviral activity against many RNA viruses including flaviviruses [44]. Treatment of ZIKV MR766-infected hPSC-HLCs and Huh7 cells with 7DMA resulted in a concentration-dependent reduction of viral replication (as assessed by quantifying viral RNA levels in the culture supernatant and in the cellular lysates). However, viral replication was not completely inhibited as the virus continued to replicate to some extent over time, both in hPSC-HLCs infected with a high and a low inoculum (Fig 2A and 2B and S2C Fig). Despite the fact that 2’CMC and T705 inhibited ZIKV replication in Huh7 cells, both antivirals failed to inhibit replication in ZIKV MR766-infected hPSC-HLCs (S2A and S2B Fig). The inhibitory effect of 7DMA on ZIKV replication was further confirmed in hESC-HLCs, infected with the PRVABC59 strain. Although, the PRVABC59 strain seemed initially to be inhibited more efficiently by 7DMA treatment than the MR766 strain, 7DMA was also unable to fully inhibit the replication of the PRVABC59 strain. Thus, although endowed with an antiviral effect, 7DMA is not considered a potent inhibitor since it only delays ZIKV replication.

### ZIKV-infected hPSC-HLCs produce infectious virions

**Fig 3:**
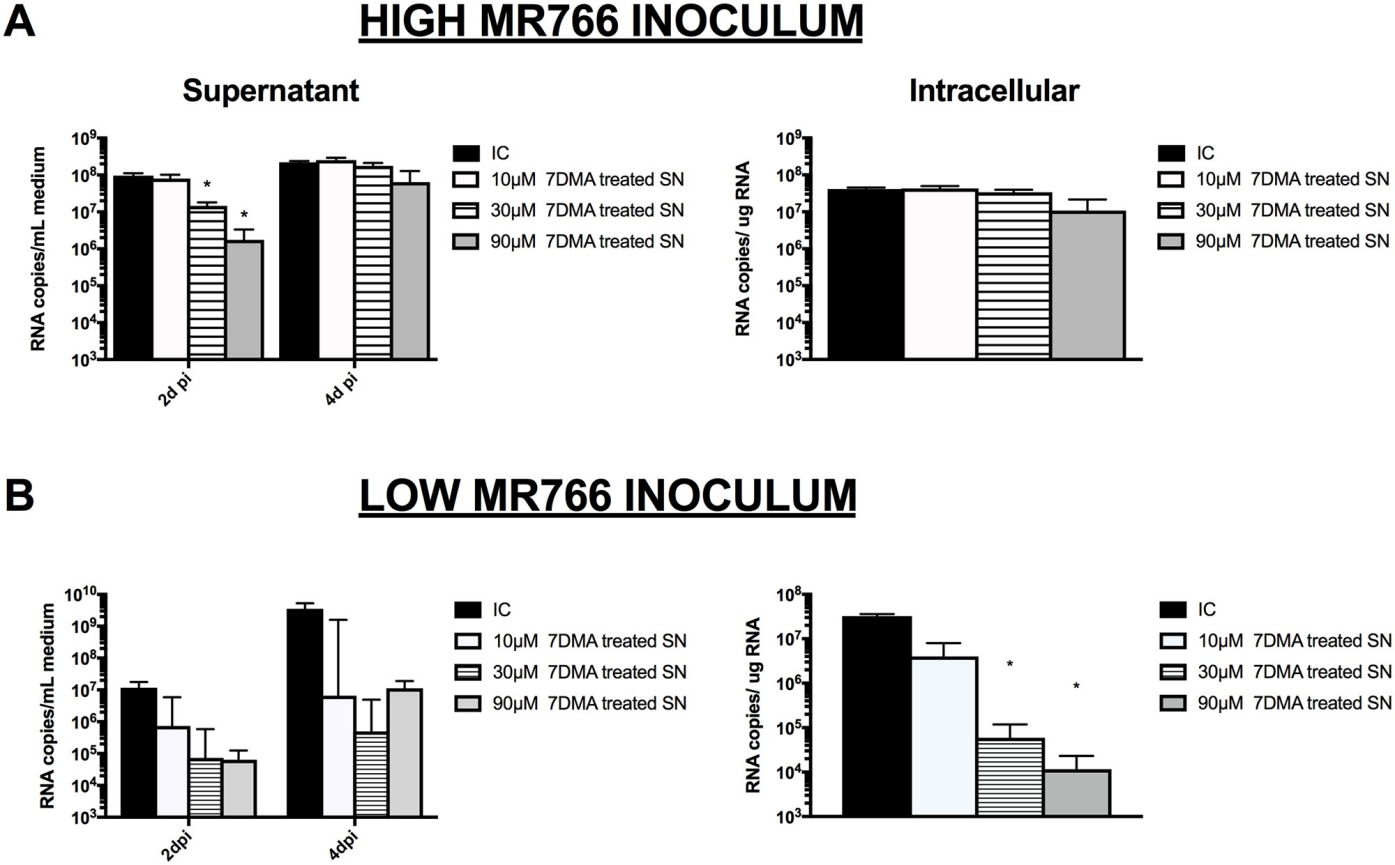
ZIKV-infected hPSC-HLCs produced infectious virions. hPSC-HLCs were re-infected with supernatant of hPSC-HLCs infected with ZIKV MR766, either treated or not treated with 7DMA. RT-qPCR was used to quantify ZIKV RNA levels (n = 3; *: p<0.05). (A) Primary cultures were inoculated with high MR766 ZIKV inoculum. (B) Primary cultures were inoculated with low MR766 ZIKV inoculum. All data are shown as mean±SEM.

To determine if ZIKV-infected hPSC-HLCs support the production of infectious ZIKV virions, supernatant from HLCs, infected with a high and low titer inoculum of MR766, was collected on 6d pi and used to re-infect hPSC-HLCs. ZIKV RNA was present at 2 and 4d pi in the supernatant and at 4d pi in the cellular lysates of secondary infected hPSC-HLC cultures (Fig 3). This demonstrates that ZIKV-infected hPSC-HLCs were able to produce new virions that were infectious as was additionally confirmed by plaque assays (S3 Fig). At 2d pi, the supernatant of 7DMA-treated primary infected cultures contained less virus particles, since lower ZIKV RNA levels were detected in the medium of secondary infected hPSC-HLCs cultures than in the medium of secondary cultures infected supernatant from untreated, infected cells. However, this effect had disappeared on 4d pi in the case of a secondary infection with supernatant from HLCs infected with a high MR766 inoculum. This initial drop in ZIKV RNA levels might also partially be explained by a carry-over effect of 7DMA from the primary cultures. In secondary infections with supernatant from HLCs infected with a low MR766 inoculum, treatment of the primary cultures with 7DMA resulted in a larger drop in the viral RNA load in the cellular lysates (Fig 3B) and, thus, a larger inhibition of virion formation in the secondary infected cultures.

### ZIKV induces a cytopathic effect in hPSC-HLCs and Huh7 cells

**Fig 4:**
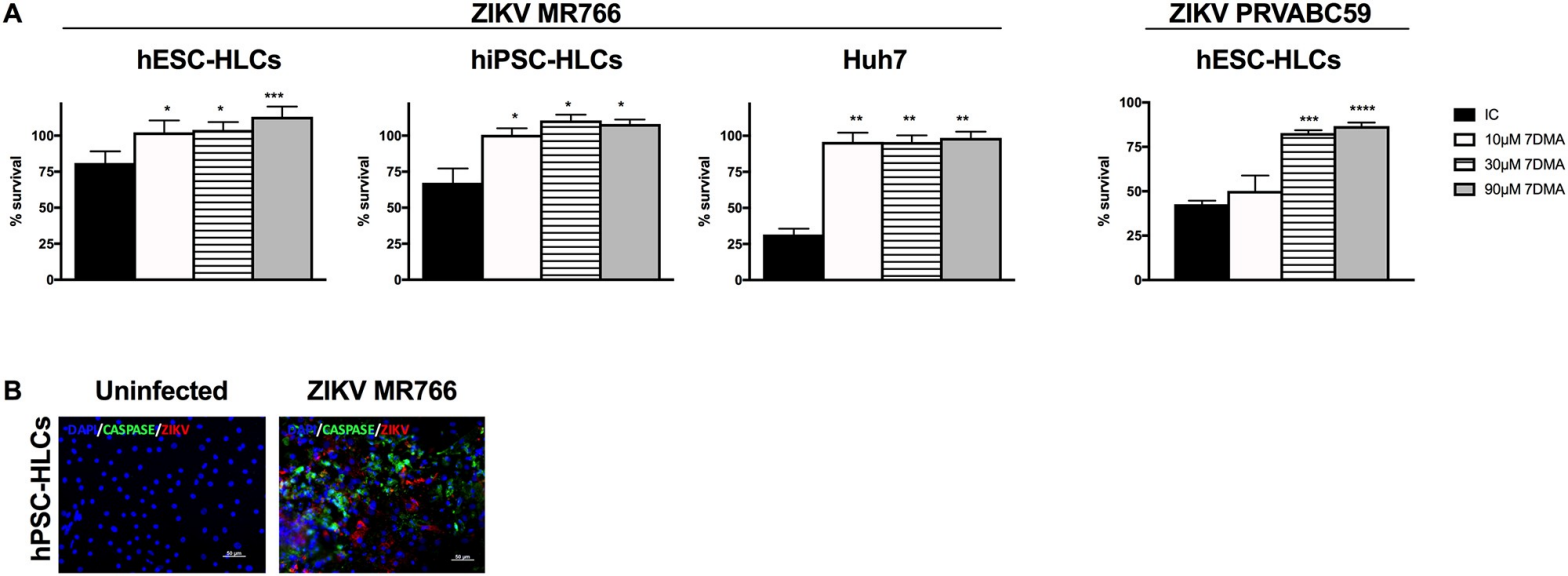
ZIKV induced a cytopathic effect in hPSC-HLCs and Huh7 cells. (A) *Left panels*: hPSC-HLCs and Huh7 cells were infected with a high MR766 inoculum. The cytopathic effect (CPE) was quantified 6d pi by MTS (3-(4,5-dimethylthiazol-2-yl)-5-(3-carboxymethoxyphenyl)-2-(4-sulfophenyl)-2*H*-tetrazolium/phenazinemetho-sulfate) readout assay. Cells were either untreated (black bars) or treated with 7DMA (10-30-90μM; remaining bars). (n = 3; *: p<0.05). *Right panel*: MTS assay was used to quantify the % survival upon infection with PRVABC59 ZIKV. Infected cells were either untreated or treated with 7DMA at different concentrations (n = 3; *: p<0.05). (B) Immunofluorescence staining for active Caspase-3 (cleaved form) in MR766 ZIKV infected hPSC-HLCs. Images are representative of three independent experiments (Scale bar = 50μM). All data are shown as mean±SEM.

ZIKV-induced cytopathic effect (CPE) in hPSC-HLCs and Huh7 cells was determined on 4d pi by measuring the cell viability staining (MTS assay) and by active Caspase-3 immunofluorescence staining. Six days post ZIKV MR766 infection, 20–30% CPE was observed in hPSC-HLCs versus 70% CPE in Huh7 cells (Fig 4A). CPE in ZIKV MR766-infected hPSC-HLCs was also confirmed by staining for active (cleaved) Caspase-3 (Fig 4B). In hPSC-HLCs, treatment with 7DMA (but not with 2’CMC nor T705) resulted in a concentration-dependent suppression of virus-induced CPE (as observed for both of the Asian and African ZIKV strains) (Fig 4A and S4A and S4B Fig). In contrast, ZIKV-induced CPE in Huh7 cells was inhibited by 7DMA (Fig 4A) as well as by 2’CMC and T705 (S4A Fig). This was not associated with an adverse metabolic effect of the compounds since none of the compounds were found to be toxic at the tested concentrations (S4C Fig).

### ZIKV induces a host innate immune response in hPSC-HLCs, but not in Huh7 cells

**Fig 5:**
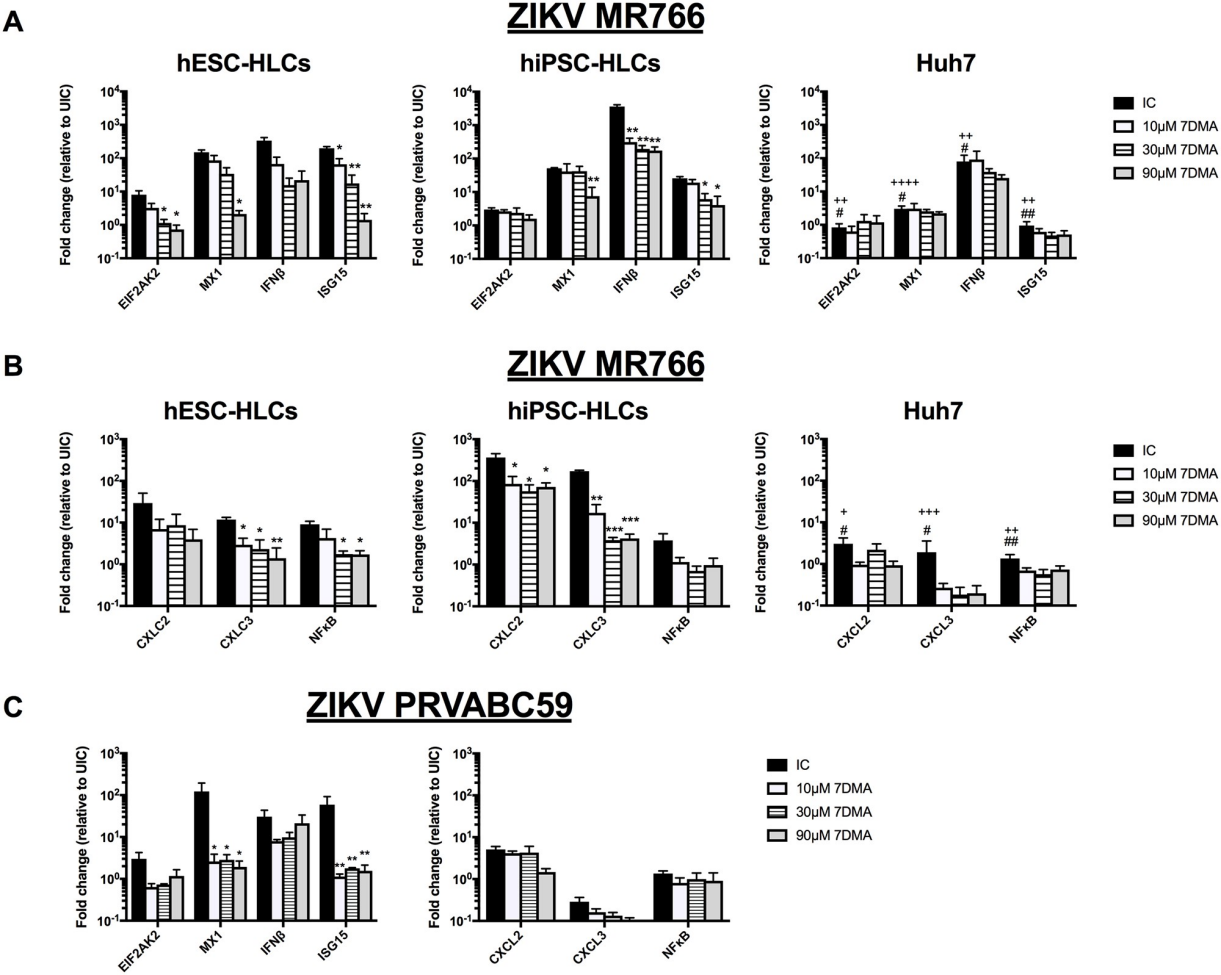
ZIKV induced an innate immune response in hPSC-HLCs, but not in Huh7 cells. (A) RT-qPCR analysis for different ISGs in high MR766 inoculum-infected hESC-HLCs, hiPSC-HLCs and Huh7. (IC = infected cells) (n = 3; * significance of treated cells to IC; + significance of infected Huh7 to infected hESC-HLCs; # significance of infected Huh7 to infected hiPSC-HLCs). (B) RT-qPCR analysis for *NFκB*, *CXLC2* and *CXCL3* in high MR766 inoculum-infected hESC-HLCs, hiPSC-HLCs and Huh7. (IC = infected cells) (n = 3; * significance of treated cells to IC; + significance of infected Huh7 to infected hESC-HLCs; # significance of infected Huh7 to infected hiPSC-HLCs). (C) RT-qPCR analysis for different ISGs and *NFκB*, *CXLC2* and *CXCL3* in hESC-HLCs infected with PRVABC59 ZIKV. (IC = infected cells) (n = 3; * significance of treated cells to IC). All data are shown as mean±SEM.

Expression of interferon stimulated genes (ISGs) is triggered upon ZIKV infection in human dermal fibroblasts and epidermal keratinocytes [45]. Moreover, the virus was recently demonstrated to target the human innate immune response by phosphorylation of STAT1 and STAT2; two transcriptional activators of ISGs [16,17,46–48]. Therefore, transcription levels of a number of ISGs (*EIF2AK2*, *MX1*, *IFN*β and *ISG15*) were quantified to examine the host immune response of ZIKV-infected cells (Fig 5A). All ISGs were found to be significantly more upregulated in hPSC-HLCs infected with MR766 strain than in Huh7 cells infected with the same strain. Moreover, in ZIKV-infected hPSC-HLCs, ISG expression was significantly and dose-dependently downregulated upon treatment with 7DMA, but not by 2’CMC or T705 treatment, whereas neither 7DMA, 2’CMC nor T705 affected ISG expression in Huh7 cells infected with the MR766 strain (Fig 5A and S5A and S5C Fig). Similarly, upon infection of hESC-HLCs with the PRVABC59 strain, ISGs were also significantly upregulated (Fig 5C) and 7DMA treatment significantly downregulated ISG expression in these infected cells. Although the infection rate and replication efficiency of the MR766 strain in hPSC-HLCs and Huh7 cells was comparable (Fig 1A and 1B), the impaired innate immunity of the Huh7 cells might explain the lower survival rate of the cells compared to ZIKV-infected hPSC-HLCs (80% for HLCs vs. 20% for Huh7 cells) (Fig 4A).

Additionally, we also assessed the induction of the NF*κ*B stress response triggered by ZIKV infection. Consistent with the induction of ISGs, a significant induction of *NFκB* and its target genes, *CXCL2* and *CXLC3*, was observed in ZIKV MR766-infected hPSC-HLCs (Fig 5B and S5B and S5D Fig), which was significantly suppressed by 7DMA treatment. Although ZIKV induced more readily CPE in Huh7 cells than in hPSC-HLC (Fig 4A), only minimal induction of *NFκB* was noted in the hepatoma cells, which also did not respond to the effects of the antiviral compounds (Fig 5B and S5B Fig). Surprisingly, *NFκB* and its target genes were not upregulated in hESC-HLCs infected with the PRVABC59 strain (Fig 5C). The lack of *NFκB* induction by PRVABC59 could explain the increased ZIKV RNA levels and increased cell apoptosis seen in hPSC-HLCs, indicative of a more infectious nature of the PRVABC59 strain.

The innate immunity response of Huh7.5 cells, a derivative clone of Huh7 cells, was previously shown to be more impaired than that of Huh7 cells when both cells were infected with HCV, making these cells highly permissive for this virus [49]. To study the potentially superior permissiveness of Huh7.5 cells for ZIKV, we performed a comparative experiment in which both Huh cells lines were infected with a high inoculum of the ZIKV MR766 strain. ZIKV RNA levels in culture supernatant and cellular lysates were quantified at different days pi. Additionally, transcription levels of the ISGs and *NFκB* and its target genes were determined to examine the innate immune and NF*κ*B stress response of ZIKV-infected Huh7.5 cells. In contrast to HCV, no significant difference was observed in infection efficiency between ZIKV-infected Huh7 cells and its derivative Huh7.5 clone (S5E Fig). Upon ZIKV infection, *IFN*β expression was slightly more upregulated in Huh7 cells than in the ZIKV-infected Huh7.5 cells. This difference, however, was not significant and did not result the expression of the downstream regulated ISGs: *EIF2AK2*, *MX1* and *ISG15*. Furthermore, no significant difference was observed in the *NFκB* stress response, which was not induced in both cell lines (S5F Fig). These results indicate that both Huh cell lines appear to be equally permissive to ZIKV.

## Discussion

The differentiation potential of human pluripotent stem cells is an interesting tool to study the tissue tropism of viruses *in vitro*. Knowledge about the tissue tropism of ZIKV is, however, very limited. Recent studies demonstrated that hiPSC-derived neuroprogenitors are susceptible to ZIKV [9–12]. When interferon-deficient mice or non-human primates were infected with ZIKV, high viral titers of ZIKV were not only found in the brain but also in the testes, spleen, kidney and liver [16,18–22,46]. Also, signs of hepatitis were observed in ZIKV-infected non-human primates [21]. Moreover, ZIKV belongs to the family of *Flaviviridae*, which also includes dengue virus, yellow fever virus and hepatitis C virus; all known to infect human hepatocytes [26,50]. Aside from case reports published in 1954 and in 2017 [23,24], no other cases of liver damage in ZIKV-infected individuals have been reported. Based on the findings in ZIKV infection mouse models and non-human primate models, we set out to study whether human hepatocytes are permissive to productive infection by ZIKV.

We demonstrated that both hPSC-HLCs and Huh7 cells can be productively infected with the prototype African ZIKV MR766 strain, as based on the presence of viral RNA, immunofluorescence staining for ZIKV NS3 and the production of infectious virions. In addition, hPSC-HLCs can be productively infected with the ZIKV PRVABC59 that belongs to the Asian lineage. This lineage contains virus strains responsible for the recent ZIKV infection outbreaks in South and Latin America, which have been linked to an increased risk for microcephaly in newborns [7,41]. ZIKV infection levels seen in hPSC-HLCs appeared to be higher compared to previously published ZIKV infection levels found in hPSC-neuron progeny [12]. This may suggest a higher replication potential of ZIKV in stem cell-derived hepatocytes compared to stem cell-derived neurons. This higher replication potential together with a less time-consuming differentiation protocol and with the use of hepatocytes for drug metabolisation/toxicity screens, indicates that hPSC-HLCs might be an interesting *in vitro* tool to screen for anti-ZIKV drugs.

We previously reported that 7DMA treatment delays disease progression in a ZIKV infection mouse model [16]. 7DMA dose-dependently inhibited viral replication of both the African and the Asian ZIKV strain in hPSC-HLCs and Huh7 cells. However, even at the highest concentration, 7DMA did not completely block ZIKV replication in infected hPSC-HLCs, as the supernatant from the 7DMA-treated cells still contained infectious virions. In contrast, 2’CMC and T705, previously shown to inhibit ZIKV replication in Vero cells [16], inhibited ZIKV replication only in Huh7 cells, not in hPSC-HLCs, indicating a possible difference in the compound processing capacity (i.e. uptake and/or drug metabolisation) between hPSC-HLCs and Huh7 cells (and Vero cells).

ZIKV infection caused CPE in both ZIKV-infected hPSC-HLCs and Huh7 cells. Although the percentage of ZIKV-infected cells was comparable between the hepatoma cell line and hPSC-HLCs, CPE was more pronounced in ZIKV-infected Huh7 cells. This discrepancy could be related to the absence of a functional innate immune response in the hepatoma cell line.

Hamel and colleagues reported that ZIKV infection of human dermal fibroblasts and epidermal keratinocytes triggers the expression of ISGs [45]. Furthermore, ZIKV was demonstrated to target the human innate immune response through degradation of STAT1 and STAT2; two transcriptional activators of ISGs [47,48]. Likewise, we demonstrate an increased expression of ISGs upon ZIKV infection (both MR766 and PRVABC59) of hPSC-HLCs. In ZIKV MR766-infected Huh7 cells, we failed, however, to detect a host innate immune response. Similar results were seen for the expression of *NFκB* and target genes in hPSC-HLCs and Huh7 cells infected with the MR766 strain: increased expression for *NFκB*, *CXCL2* and *CXCL3* in hPSC-HLCs, whereas no increased expression in Huh7 cells, indicating that major differences exist between the hepatoma cell line Huh7 and hPSC-HLCs. In contrast, upon ZIKV infection, no difference was observed in the innate immunity response and the induction of the NF*κ*B pathway in Huh7 cells and its derivative cell line, Huh7.5, resulting in comparable high ZIKV RNA levels in the supernatant and cellular lysates. This is in contrast to the high permissiveness of Huh7.5 for HCV as a result of a weaker innate immunity response compared to that of the Huh7 cells [49]. Finally, we observed that the expression of *NFκB* and its target genes was not induced in hPSC-HLCs infected with the PRVABC59 strain. The lack of a PRVABC59-mediated induction of *NFκB* has also been observed by others [51,52] and could explain the increased ZIKV replication and cell death seen in hPSC-HLCs infected with the PRVABC59 strain compared to hPSC-HLCs infected with MR766 ZIKV. Studies that aim to understand the different *NFκB* response induced by the MR766 and PRVABC59 strains might be of interest to gain insights in how to combat the more virulent PRVABC59 ZIKV strain.

## Conclusion

In conclusion, hepatoma cells as well as hPSC-HLCs are highly susceptible to ZIKV infection *in vitro*. The antiviral effect of three viral polymerase inhibitors and the host immune response upon infection were, however, significantly different between ZIKV-infected Huh7 cells and hPSC-HLCs. As hPSC-HLCs more closely resemble primary human hepatocytes (PHHs) compared to transformed Huh7 cells, our study demonstrates the need for assessing the effect of antiviral drugs, not only in cancerous (e.g. hepatoma cells) and immortalized (e.g. Vero cells) cell lines, but also in more physiologically relevant *in vitro* systems. Moreover, we provide compelling evidence that, in line with the observed ZIKV infection in the hepatocytes of mice and non-human primates, ZIKV can also infect human hepatocytes. It should hence be further explored whether ZIKV may infect the liver, in particular in the developing fetus.

## Supporting information

S1 Fig hPSC were differentiated towards HLCs and are highly susceptible to ZIKV infection. (A) Immunofluorescence staining of d20 hPSC-HLCs for hepatocyte markers AFP and ALB. (B) Immunofluorescence staining of d20 hPSC-HLCs for hepatocyte markers HNF4α and NTCP. (C) Albumin secretion by hPSC-HLCs at d20 of differentiation. (D) RT-qPCR analysis of the supernatant and cellular lysates (intracellular) of hESC-HLCs, hiPSC-HLCs and Huh7 infected with a low MR766 inoculum (d pi = days post infection) (n = 3). All data are shown as mean±SEM.
(TIFF)

S2 Fig 2’CMC and T705 did not inhibit ZIKV replication in hPSC-HLCs, unlike in Huh7 cells. (A) RT-qPCR analysis of the supernatant of a high MR766 inoculum-infected hESC-HLCs, hiPSC-HLCs and Huh7 cells. Infected cells (IC) were treated with increasing concentrations of 2’CMC (5μM—45μM) or T705 (25μM—225μM) (n = 3; *: p<0.05). (B) RT-qPCR analysis of the cellular lysates (intracellular) of hESC-HLCs, hiPSC-HLCs and Huh7 cells infected with the high MR766 inoculum. Infected cells were treated with increasing concentrations of 2’CMC (5μM—45μM) or T705 (25μM—225μM) (n = 3; *: p<0.05). (C) RT-qPCR analysis of the supernatant of hPSC-HLCs infected with a low MR766 inoculum. Infected cells (IC) were treated with increasing concentrations of 7DMA (10μM—90μM) (n = 3; *: p<0.05). All data are shown as mean±SEM.
(TIFF)

S3 Fig Plaque assay with MR766 ZIKV demonstrated the formation of infectious virions by hPSC-HLCs infected cells. (A) Baby Hamster Kidney (BHK) cells were inoculated with 6d pi supernatant from hESC-HLCs, infected with high or low ZIKV MR766 inoculum. The inoculum was diluted 1:10–1:1250.
(TIFF)

S4 Fig 2’CMC and T705 did not inhibit CPE in hPSC-HLCs, while they did inhibit CPE in Huh7 cells. (A) hPSC-HLCs and Huh7 cells were infected high MR766 inoculum. CPE was quantified by MTS readout. Cells were either untreated (IC = infected cell) or treated with 2’CMC or T705 (n = 3; *p = 0.05). (B) hPSC-HLCs and Huh7 cells were infected with the PRVABC59 clinical isolate. CPE was quantified by MTS readout. Cells were either untreated (IC = infected cell) or treated with 2’CMC or T705 (n = 3; *p = 0.05). (C) hPSC-HLCs were either untreated (control) or treated with different concentrations of 7DMA, 2’CMC or T705. Compound toxicity was quantified by MTS readout (n = 3). All data are shown as mean±SEM.
(TIFF)

S5 Fig ZIKV induced an innate immune and NFκβ response in infected hPSC-HLCs, not in infected Huh7 cells. (A) hPSC-HLCs and Huh7 cells were infected with a high MR766 inoculum and treated with either 2’CMC or T705. RT-qPCR analysis for different ISGs. (IC = infected cell) (n = 3; * significance of treated cells to IC; + significance of IC Huh7 to IC hESC-HLCs; # significance of IC Huh7 to hiPSC-HLCs). (B) hPSC-HLCs and Huh7 cells were infected with a high MR766 inoculum and treated with either 2’CMC or T705. RT-qPCR analysis for *NFκβ* and downstream regulated genes. (IC = infected cell) (n = 3; * significance of treated cells to IC; + significance of IC HuH7 to IC hESC-HLCs; # significance of IC Huh7 to hiPSC-HLCs). (C) hPSC-HLCs and Huh7 cells were infected with a low MR766 inoculum and treated with 7DMA. RT-qPCR analysis for different ISGs. (IC = infected cell) (n = 3; * significance of treated cells to IC). (D) hPSC-HLCs and Huh7 cells were infected with a low MR766 inoculum and treated with 7DMA. RT-qPCR analysis for *NFκβ* and downstream regulated genes. (IC = infected cell) (n = 3; * significance of treated cells to IC). (E) ZIKV infection of Huh7 and Huh7.5 cells using a high ZIKV MR766 inoculum. RT-qPCR analysis was performed to quantify viral RNA levels in the supernatant and cellular lysates (intracellular) (d pi = days post infection) (n = 3). (F) RT-qPCR analysis for different ISGs and *NFκβ* and its downstream regulated genes in Huh7 and Huh7.5 cells infected with a high inoculum of ZIKV MR766. All data are represented as mean±SEM.
(TIFF)

S1 Table Primer list. (PDF)

S2 Table List of antibodies. (PDF)
