# Supplementary figures and images for: Human stem cell-derived hepatocyte-like cells support Zika virus replication and provide a relevant model to assess the efficacy of potential antivirals
Source: PLoS One. 2018 Dec 19;13(12):e0209097. doi: 10.1371/journal.pone.0209097 (PMC6300258; doi:10.1371/journal.pone.0209097)

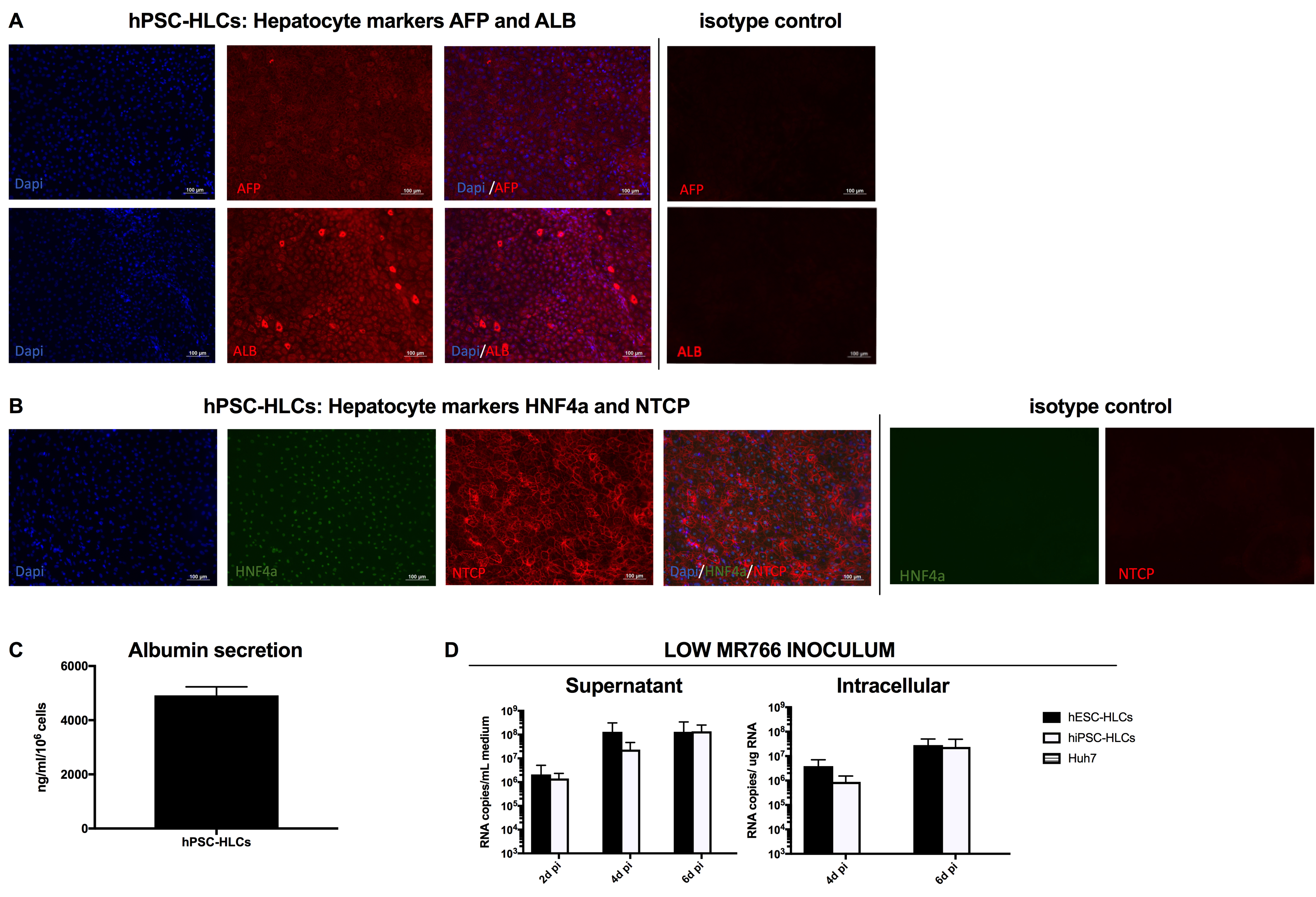

Supplement: S1 Fig — (A) Immunofluorescence staining of d20 hPSC-HLCs for hepatocyte markers AFP and ALB. (B) Immunofluorescence staining of d20 hPSC-HLCs for hepatocyte markers HNF4α and NTCP. (C) Albumin secretion by hPSC-HLCs at d20 of differentiation. (D) RT-qPCR analysis of the supernatant and cellular lysates (intracellular) of hESC-HLCs, hiPSC-HLCs and Huh7 infected with a low MR766 inoculum (d pi = days post infection) (n = 3). All data are shown as mean±SEM. (TIFF) [file pone.0209097.s001.tiff]

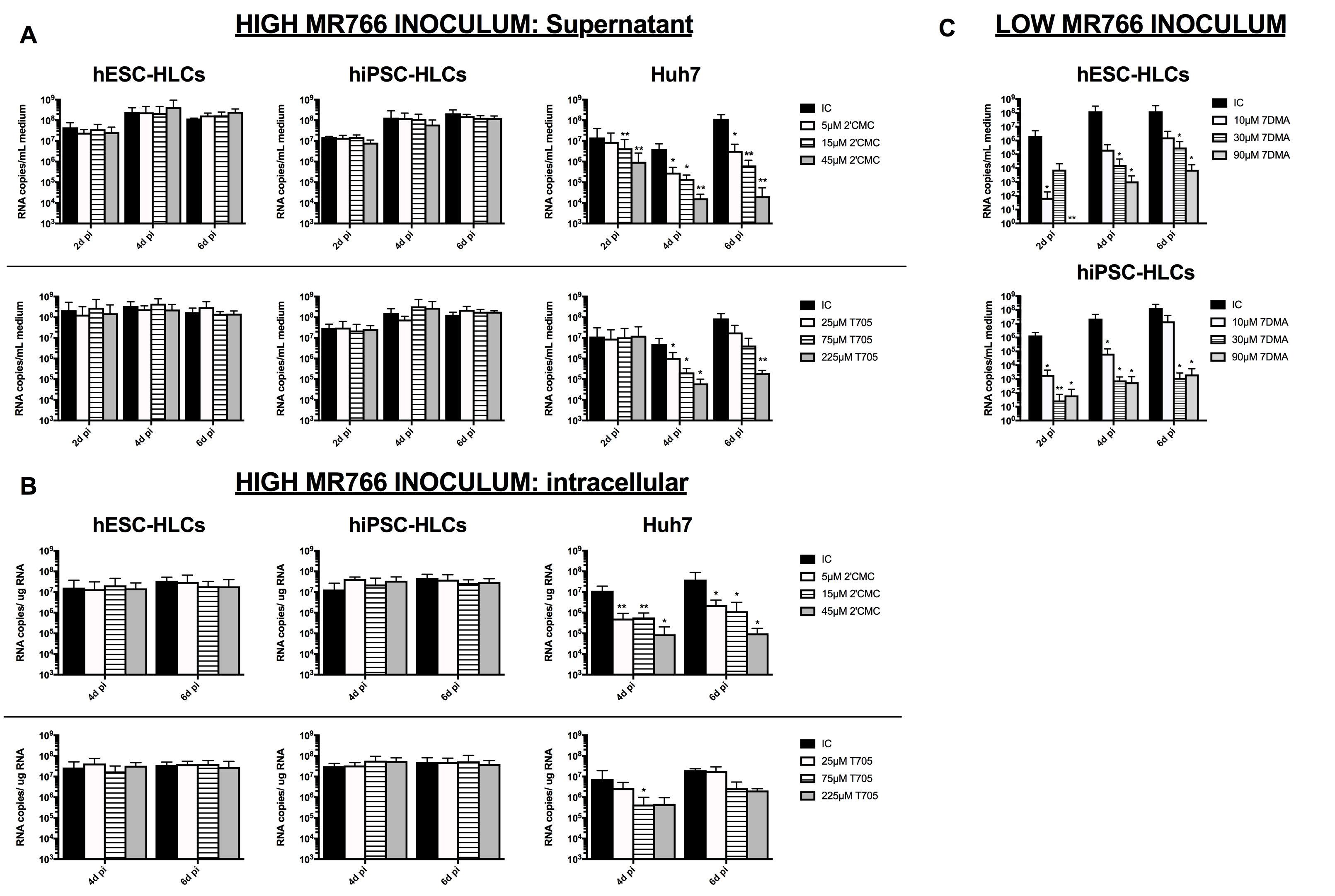

Supplement: S2 Fig — (A) RT-qPCR analysis of the supernatant of a high MR766 inoculum-infected hESC-HLCs, hiPSC-HLCs and Huh7 cells. Infected cells (IC) were treated with increasing concentrations of 2’CMC (5μM—45μM) or T705 (25μM—225μM) (n = 3; *: p<0.05). (B) RT-qPCR analysis of the cellular lysates (intracellular) of hESC-HLCs, hiPSC-HLCs and Huh7 cells infected with the high MR766 inoculum. Infected cells were treated with increasing concentrations of 2’CMC (5μM—45μM) or T705 (25μM—225μM) (n = 3; *: p<0.05). (C) RT-qPCR analysis of the supernatant of hPSC-HLCs infected with a low MR766 inoculum. Infected cells (IC) were treated with increasing concentrations of 7DMA (10μM—90μM) (n = 3; *: p<0.05). All data are shown as mean±SEM. (TIFF) [file pone.0209097.s002.tiff]

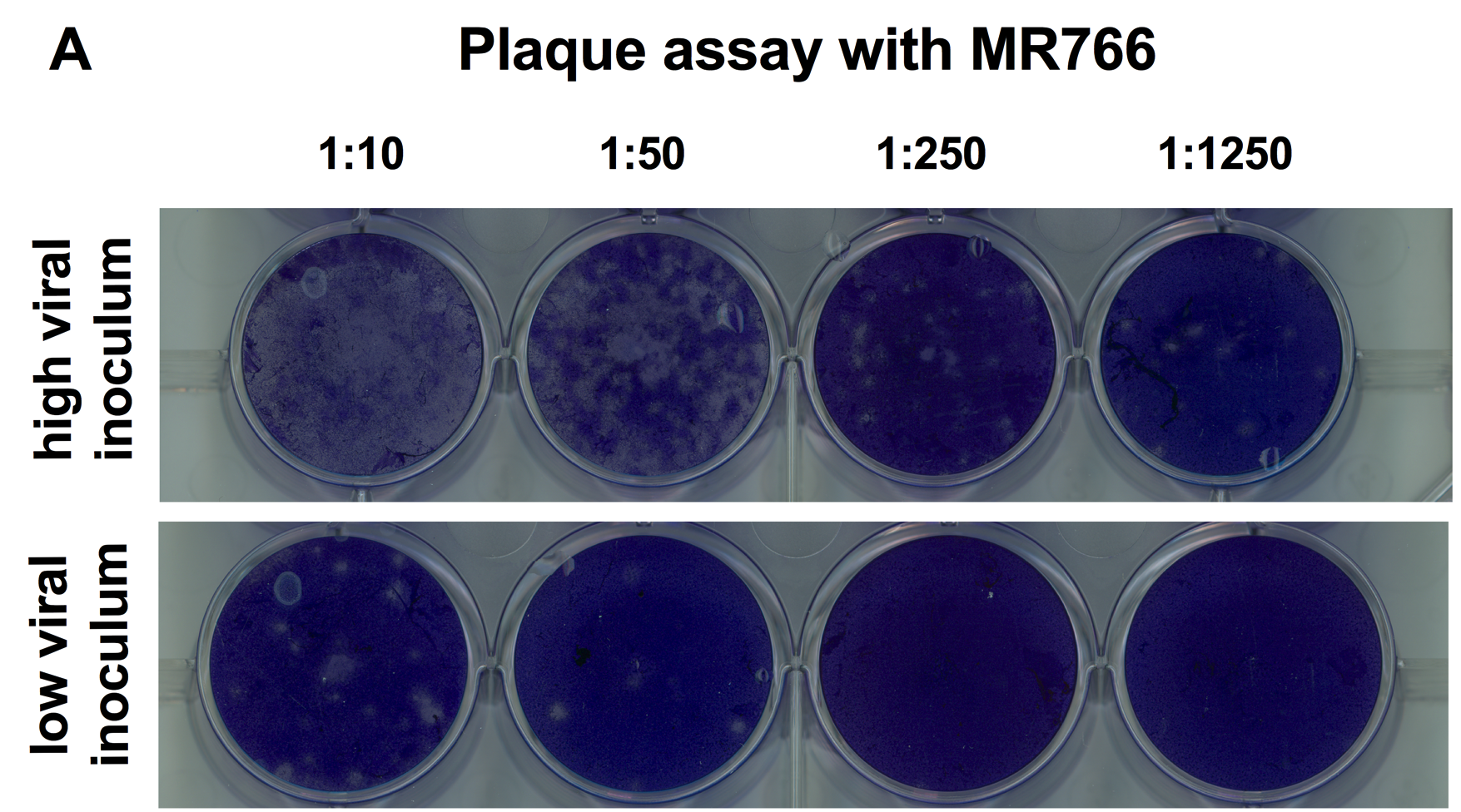

Supplement: S3 Fig — (A) Baby Hamster Kidney (BHK) cells were inoculated with 6d pi supernatant from hESC-HLCs, infected with high or low ZIKV MR766 inoculum. The inoculum was diluted 1:10–1:1250. (TIFF) [file pone.0209097.s003.tiff]

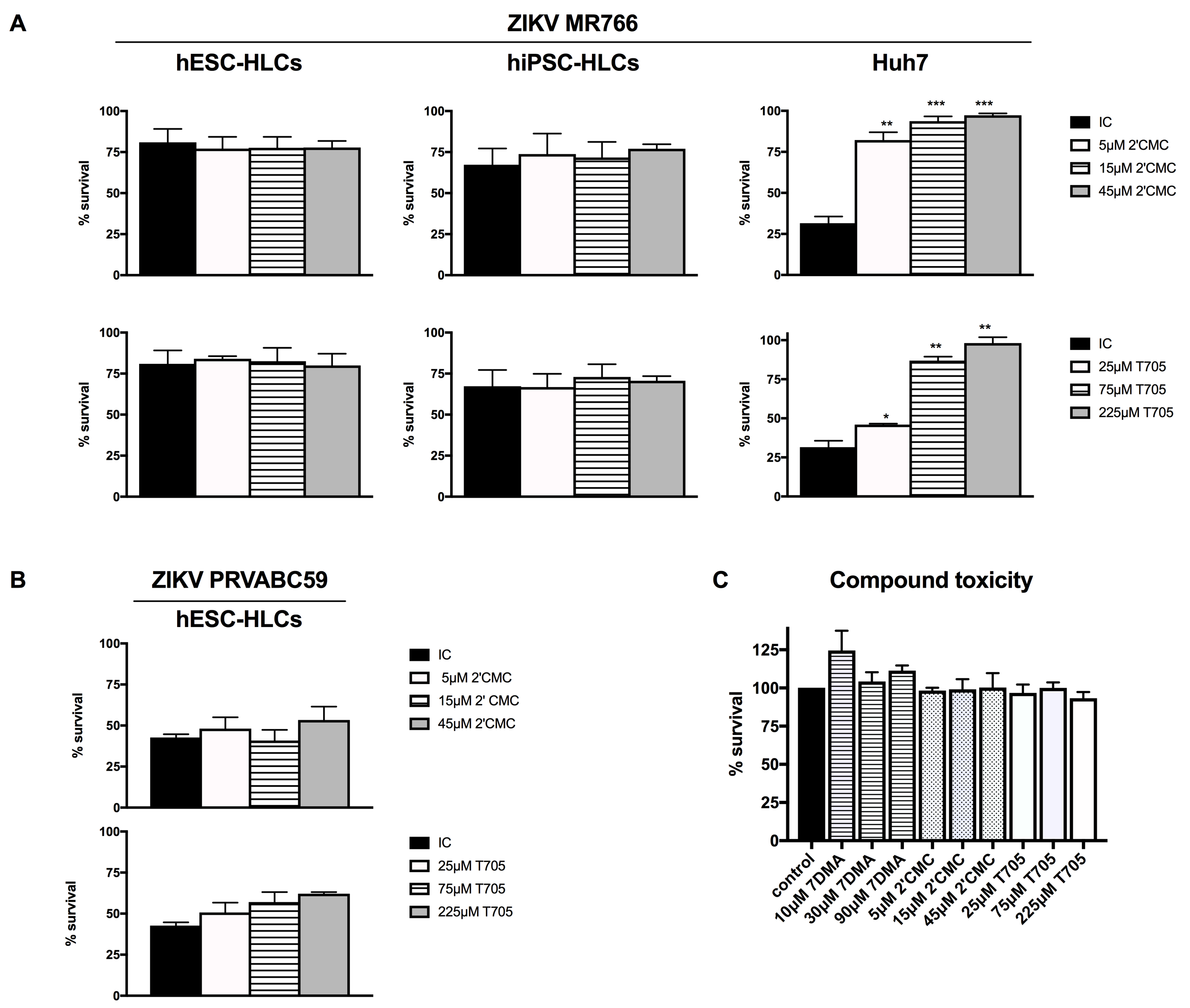

Supplement: S4 Fig — (A) hPSC-HLCs and Huh7 cells were infected high MR766 inoculum. CPE was quantified by MTS readout. Cells were either untreated (IC = infected cell) or treated with 2’CMC or T705 (n = 3; *p = 0.05). (B) hPSC-HLCs and Huh7 cells were infected with the PRVABC59 clinical isolate. CPE was quantified by MTS readout. Cells were either untreated (IC = infected cell) or treated with 2’CMC or T705 (n = 3; *p = 0.05). (C) hPSC-HLCs were either untreated (control) or treated with different concentrations of 7DMA, 2’CMC or T705. Compound toxicity was quantified by MTS readout (n = 3). All data are shown as mean±SEM. (TIFF) [file pone.0209097.s004.tiff]

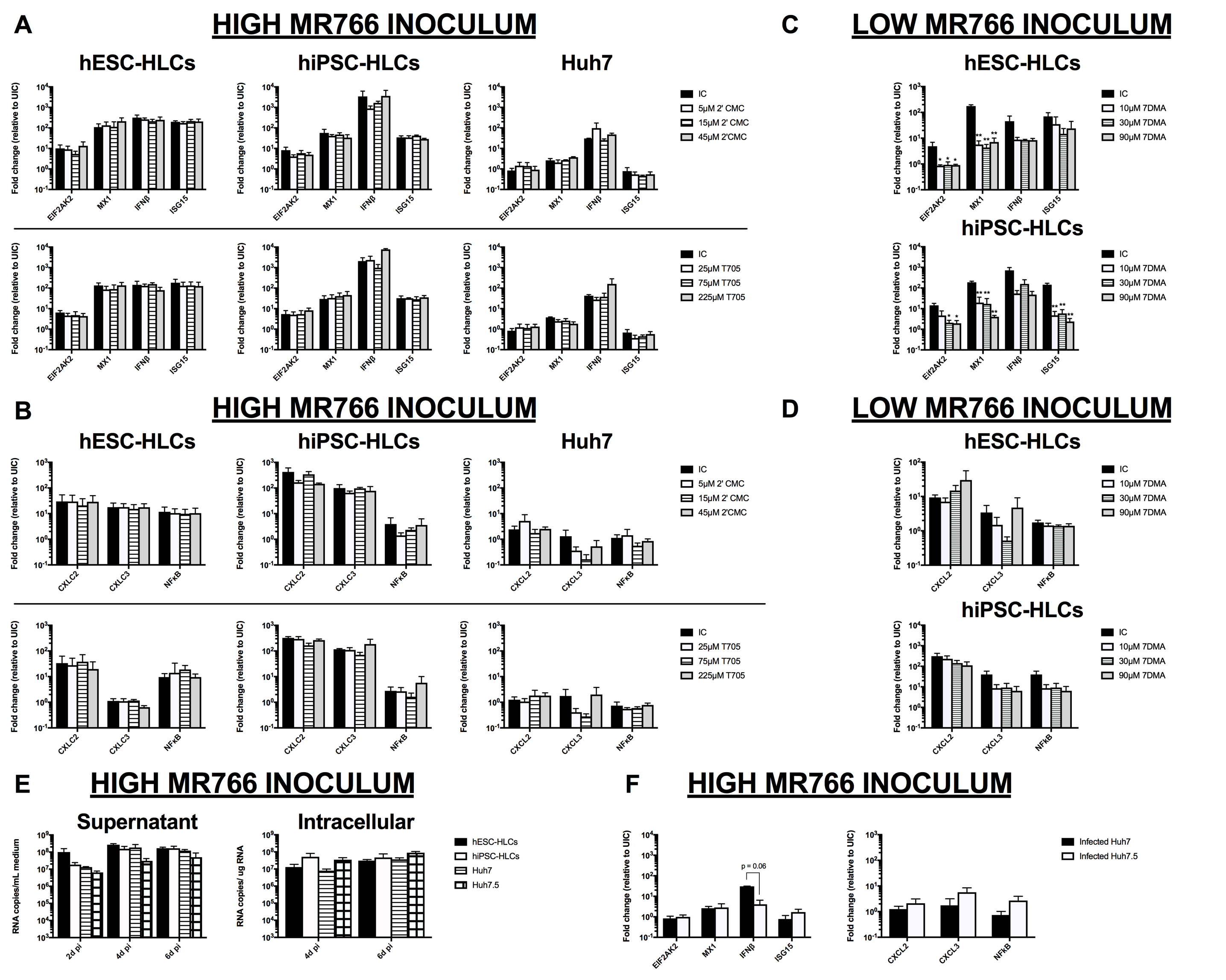

Supplement: S5 Fig — (A) hPSC-HLCs and Huh7 cells were infected with a high MR766 inoculum and treated with either 2’CMC or T705. RT-qPCR analysis for different ISGs. (IC = infected cell) (n = 3; * significance of treated cells to IC; + significance of IC Huh7 to IC hESC-HLCs; # significance of IC Huh7 to hiPSC-HLCs). (B) hPSC-HLCs and Huh7 cells were infected with a high MR766 inoculum and treated with either 2’CMC or T705. RT-qPCR analysis for NFκβ and downstream regulated genes. (IC = infected cell) (n = 3; * significance of treated cells to IC; + significance of IC HuH7 to IC hESC-HLCs; # significance of IC Huh7 to hiPSC-HLCs). (C) hPSC-HLCs and Huh7 cells were infected with a low MR766 inoculum and treated with 7DMA. RT-qPCR analysis for different ISGs. (IC = infected cell) (n = 3; * significance of treated cells to IC). (D) hPSC-HLCs and Huh7 cells were infected with a low MR766 inoculum and treated with 7DMA. RT-qPCR analysis for NFκβ and downstream regulated genes. (IC = infected cell) (n = 3; * significance of treated cells to IC). (E) ZIKV infection of Huh7 and Huh7.5 cells using a high ZIKV MR766 inoculum. RT-qPCR analysis was performed to quantify viral RNA levels in the supernatant and cellular lysates (intracellular) (d pi = days post infection) (n = 3). (F) RT-qPCR analysis for different ISGs and NFκβ and its downstream regulated genes in Huh7 and Huh7.5 cells infected with a high inoculum of ZIKV MR766. All data are represented as mean±SEM. (TIFF) [file pone.0209097.s005.tiff]
