# Supplementary material for: Human stem cell-derived hepatocyte-like cells support Zika virus replication and provide a relevant model to assess the efficacy of potential antivirals
Source: PLoS One. 2018 Dec 19;13(12):e0209097. doi: 10.1371/journal.pone.0209097 (PMC6300258; doi:10.1371/journal.pone.0209097)
Supplement: S1 Table — (PDF) [file pone.0209097.s006.pdf]

Supplementary Table 1. Primer list

Gene	Forward primer	Reverse primer
GAPDH	TCAAGAAGGTGGTGAAGCAGG	ACCAGGAAATGAGCTTGACAAA
EIF2AK2	GAGAATTTCCAGAAGGTGAAGG T	ATTCCCCATGGATAATCCTTCT
MX1	TGCTTATCCGTTAGCCGTGG	CGCCAGCTCATGTGCATCT
ISG15	GAGAGGCAGCGAACTCATCT	CTTCAGCTCTGACACCGACA
IFNβ	AAACTCATGAGCAGTCTGCA	AGGAGATCTTCAGTTTCGGAGG
CXCL2	CCACACTCAAGAATGGGCA	CAATAAGCTTCCTCCTTCCTTCTG
CXCL3	TAGCCACACTCAAGAATGGGAA	TCTCTCCTGTCAGTTGGTGC
NFκB	CAGAGAGTGAGGATGAGGAGAG	TCATCATAGGGCAGCTCGT
ZIKV NS1	TGACTCCCCTCGTAGACTG	CTCTCCTTCCACTGATTTCCAC
ZIKV E	CCGCTGCCCAACACAAG	CCACTAACGTTCTTTTGCAGACAT
