# Supplementary material for: Human stem cell-derived hepatocyte-like cells support Zika virus replication and provide a relevant model to assess the efficacy of potential antivirals
Source: PLoS One. 2018 Dec 19;13(12):e0209097. doi: 10.1371/journal.pone.0209097 (PMC6300258; doi:10.1371/journal.pone.0209097)
Supplement: S2 Table — (PDF) [file pone.0209097.s007.pdf]

Supplementary Table 2. List of antibodies

Antibody	Catalog number	Company	Dilution
HNF4a	Ab41898	Abcam	1:200
Flavivirus Group Antigen (clone D1-4G2-4-1)	MAB10216	Millipore	1:500
Cleaved Caspase 3	AB3623	Millipore	1:200
ZIKV NS3		Andres Merits Lab	1:500
AFP	A008	Dako	1:600
ALB	A0001	Dako	1:4000
NTCP	HPA042727	Sigma-Aldrich	1:500
